# Supplementary material for: Visualizing the mechanism of quinol oxidation and inhibition of a bd-type oxidase using cryo-EM
Source: Sci Adv. 2026 May 20;12(21):eaec9946. doi: 10.1126/sciadv.aec9946 (PMC13189123; doi:10.1126/sciadv.aec9946)
Supplement: Supplementary file 1 — Figs. S1 to S17 Tables S1 to S4 References [file sciadv.aec9946_sm.pdf]

Science Advances
AAAS

Supplementary Materials for

Visualizing the mechanism of quinol oxidation and inhibition of a *bd*-type oxidase using cryo-EM

Tijn T. van der Velden *et al.*

Corresponding author: Lars J. C. Jeuken, l.j.c.jeuken@lic.leidenuniv.nl

Sci. Adv. **12**, eaec9946 (2026)
DOI: 10.1126/sciadv.aec9946

This PDF file includes:

Figs. S1 to S17
Tables S1 to S4
References

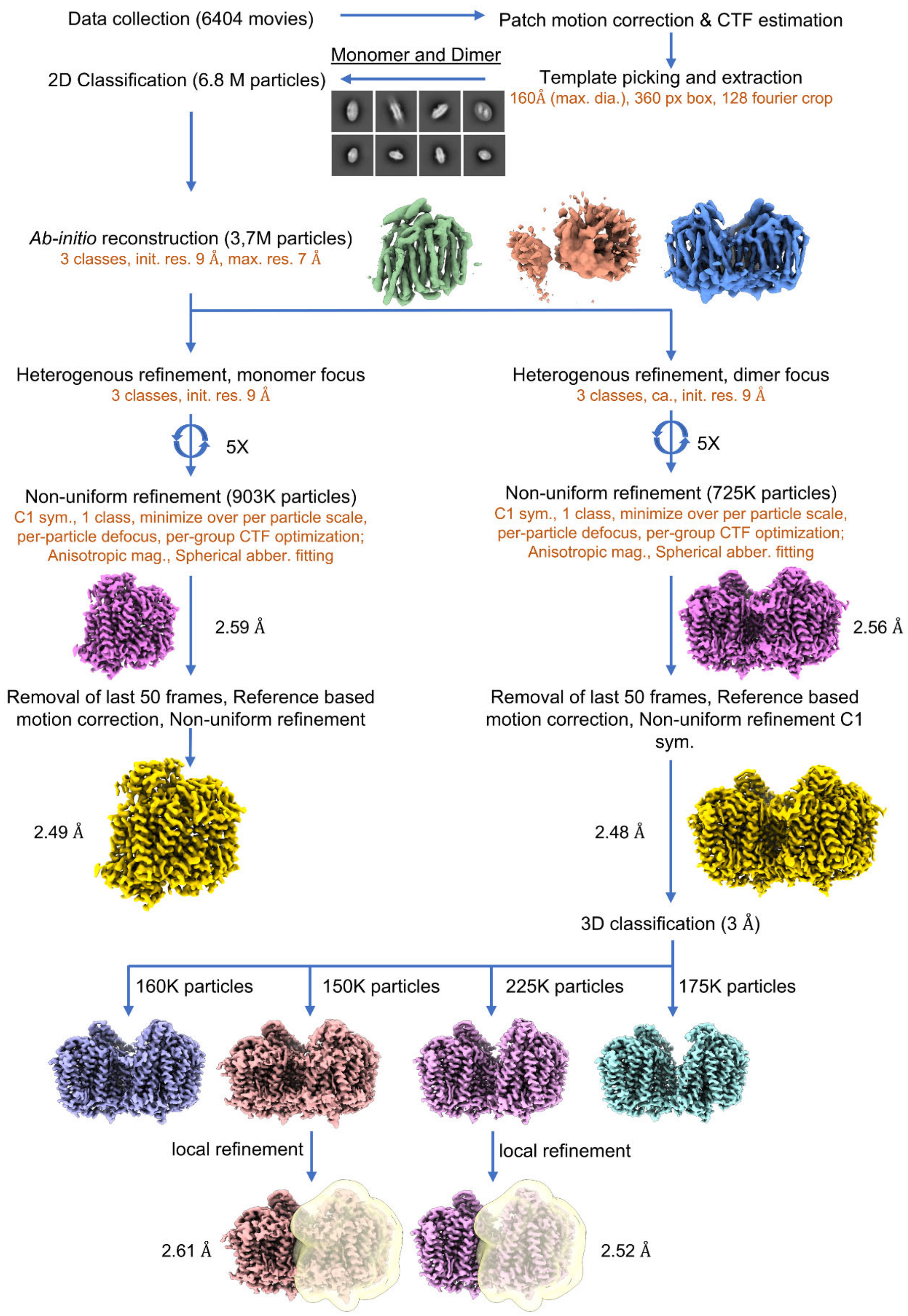

Fig. S1. Cryo EM processing of the *Ecbd* unbound and bound^{open} structures (9SE4, 9SFV, 9SFJ). Templates for template picking were generated using the MK-bound dataset (in nanodiscs). The same templates were used for processing of all other datasets.

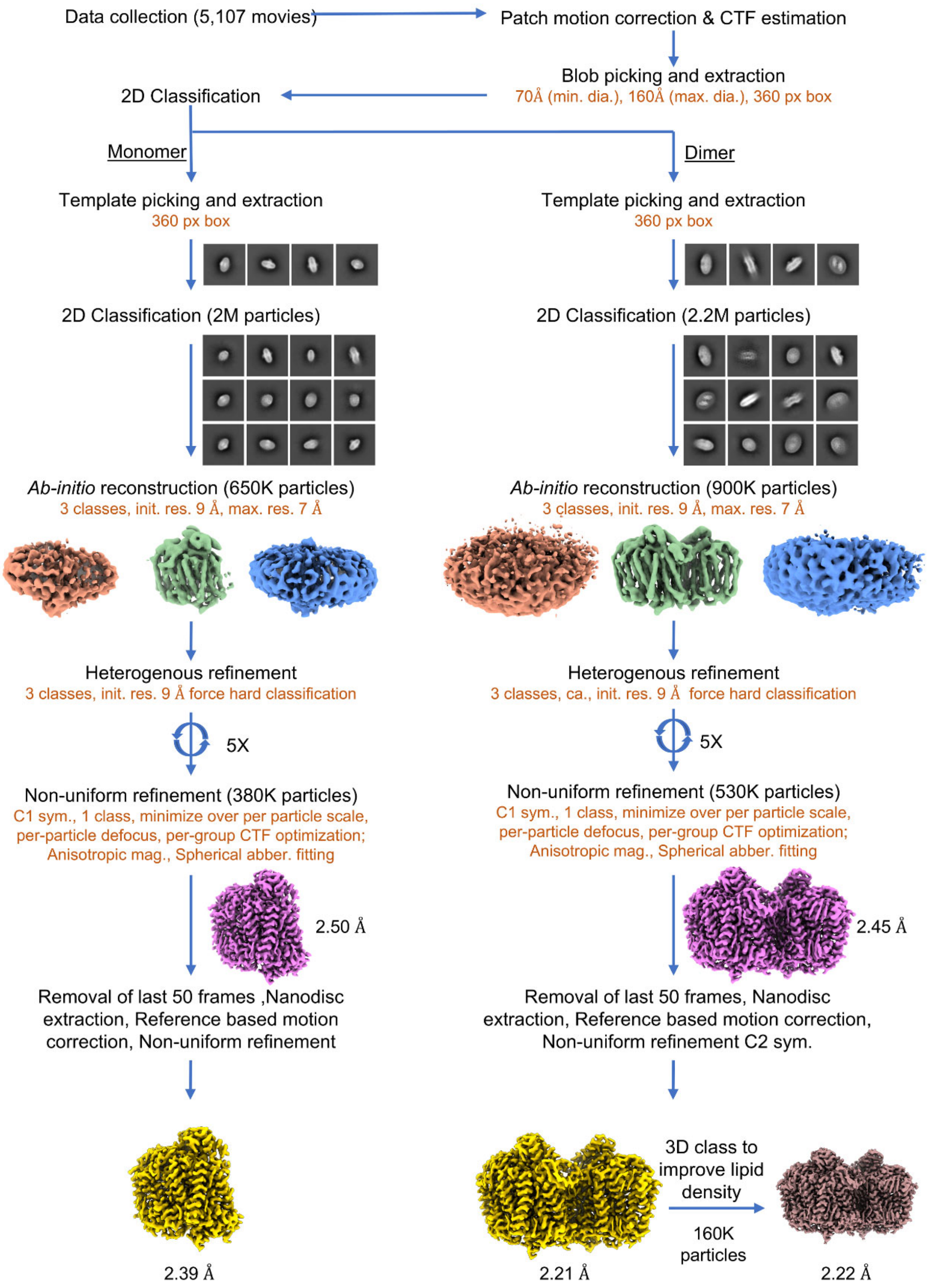

Fig. S2. Cryo-EM processing of the *Ecbd* MK bound^{closed} structure (9SFF, 9RZV). Templates for template picking were generated using the MK-bound dataset (in nanodiscs) as shown in Fig. S1. The same templates were used for processing of all other datasets.

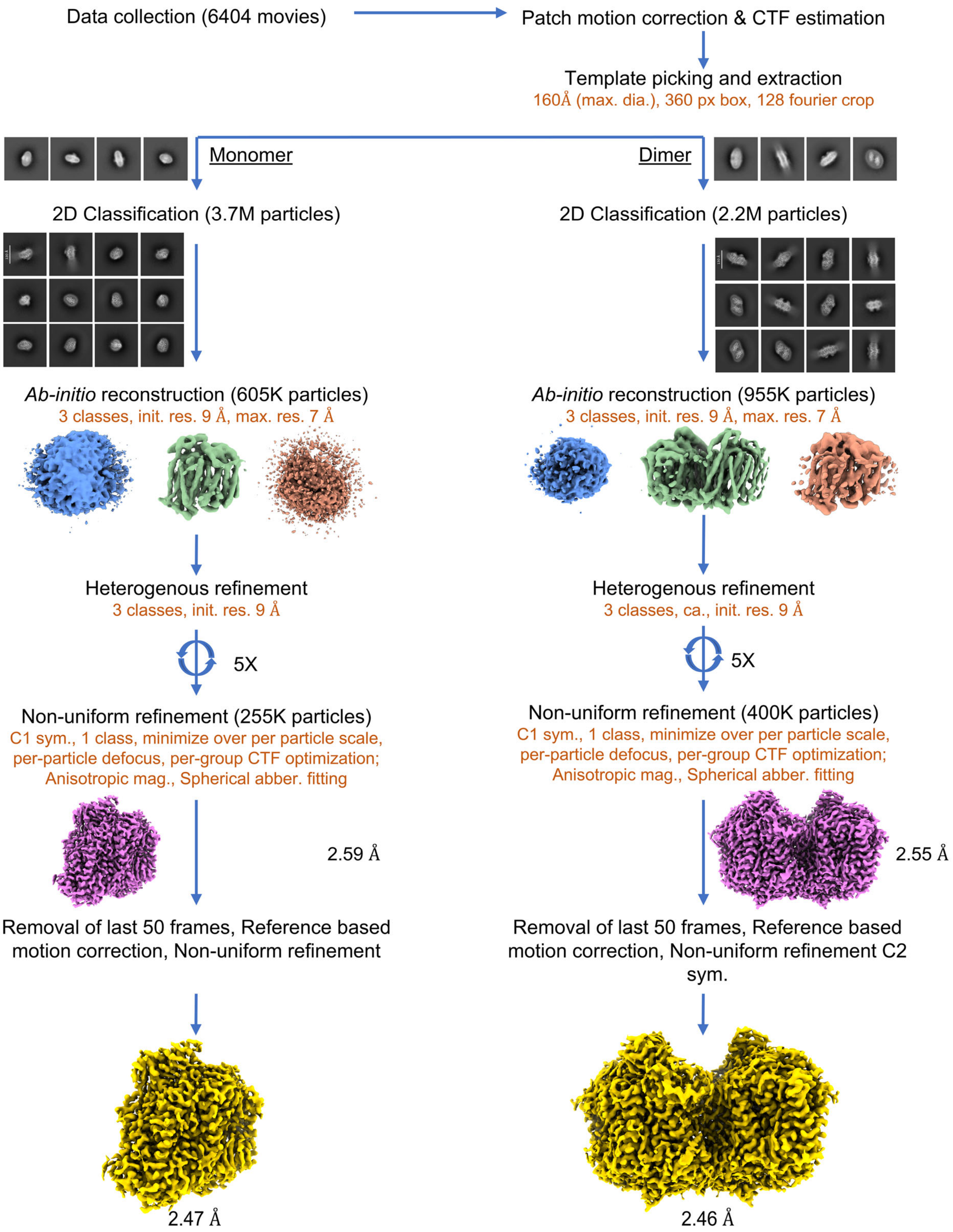

Fig. S3. Cryo-EM processing of the Aurachin D bound *Ecbd* structures (9SFH, 9SEJ). Templates for template picking were generated using the MK-bound dataset (in nanodiscs) as shown in Fig. S1. The same templates were used for processing of all other datasets.

Sample	PDB	EMDB
Ecbd monomer MK incubated	9SFF	54823

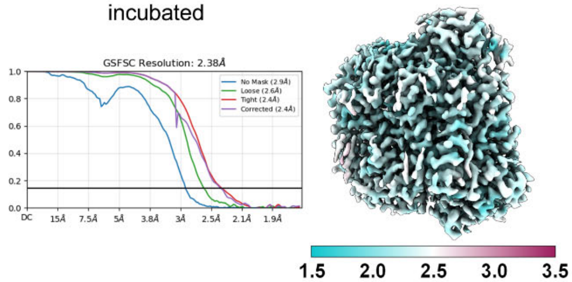

Sample	PDB	EMDB
Ecbd dimer MK bound closed	9RZV	54414

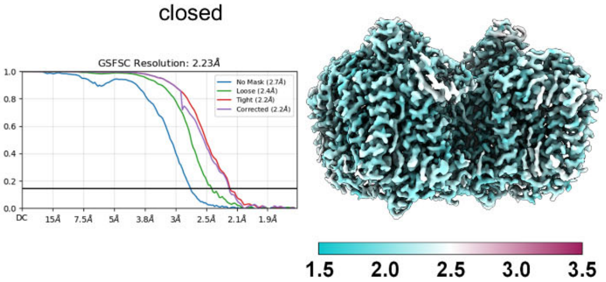

Sample	PDB	EMDB
Ecbd monomer AurD incubated	9SE4	54822

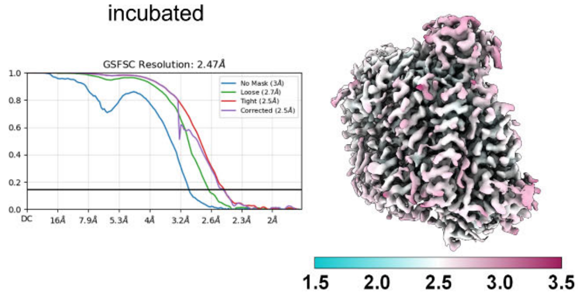

Sample	PDB	EMDB
Ecbd dimer AurD bound	9SEJ	54812

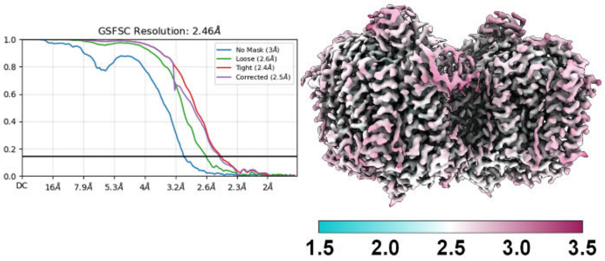

Sample	PDB	EMDB
Ecbd monomer Apo	9SFH	54823

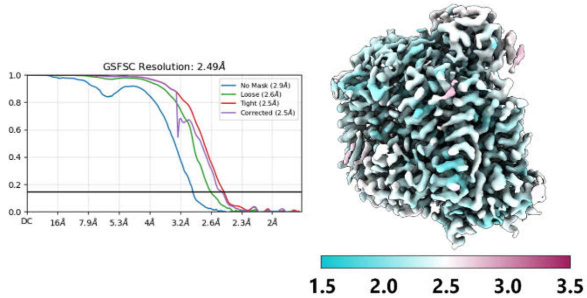

Sample	PDB	EMDB
Ecbd dimer MK bound Open/closed	9SFJ	54826

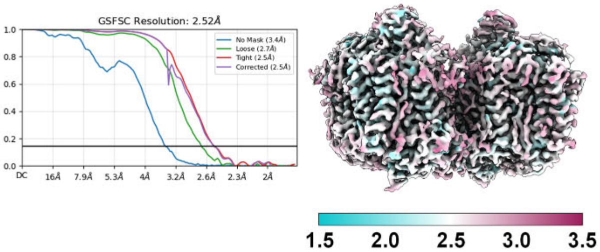

Sample	PDB	EMDB
Ecbd dimer apo/MK closed	9SFV	54866

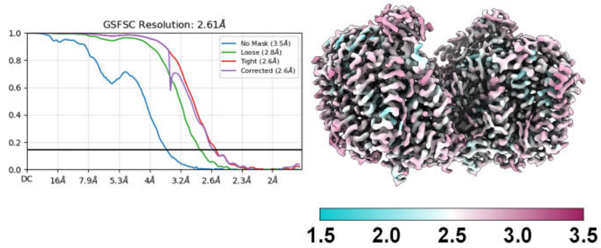

Fig. S4. GSFSC curves and local resolution estimates

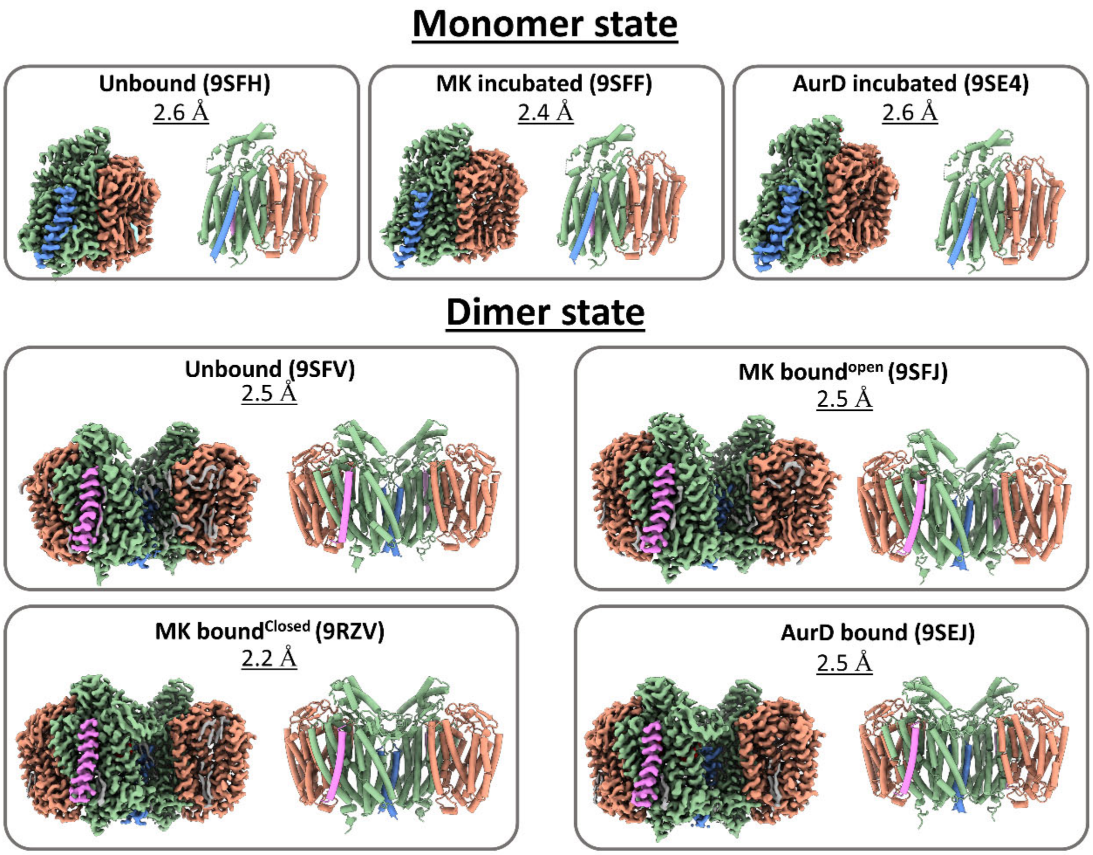

Fig. S5. Overview of cryo-EM structures solved in this study

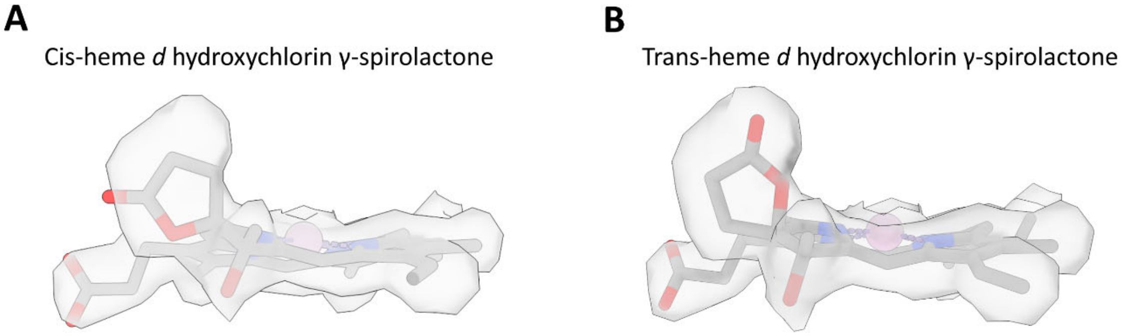

Fig. S6. Fitting of the heme density with **(A)** cis heme d hydroxychlorin γ-spirolactone or **(B)** trans heme d hydroxychlorin γ-spirolactone.

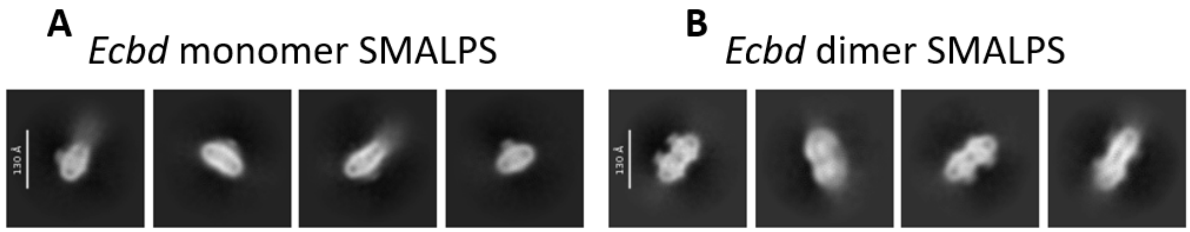

Fig. S7. 2D classes of *Ecbd* after SMALP isolation (**A**) 2D classes of the *Ecbd* monomer (**B**) 2D classes of the *Ecbd* dimer showing of both oligomeric states *in vivo*.

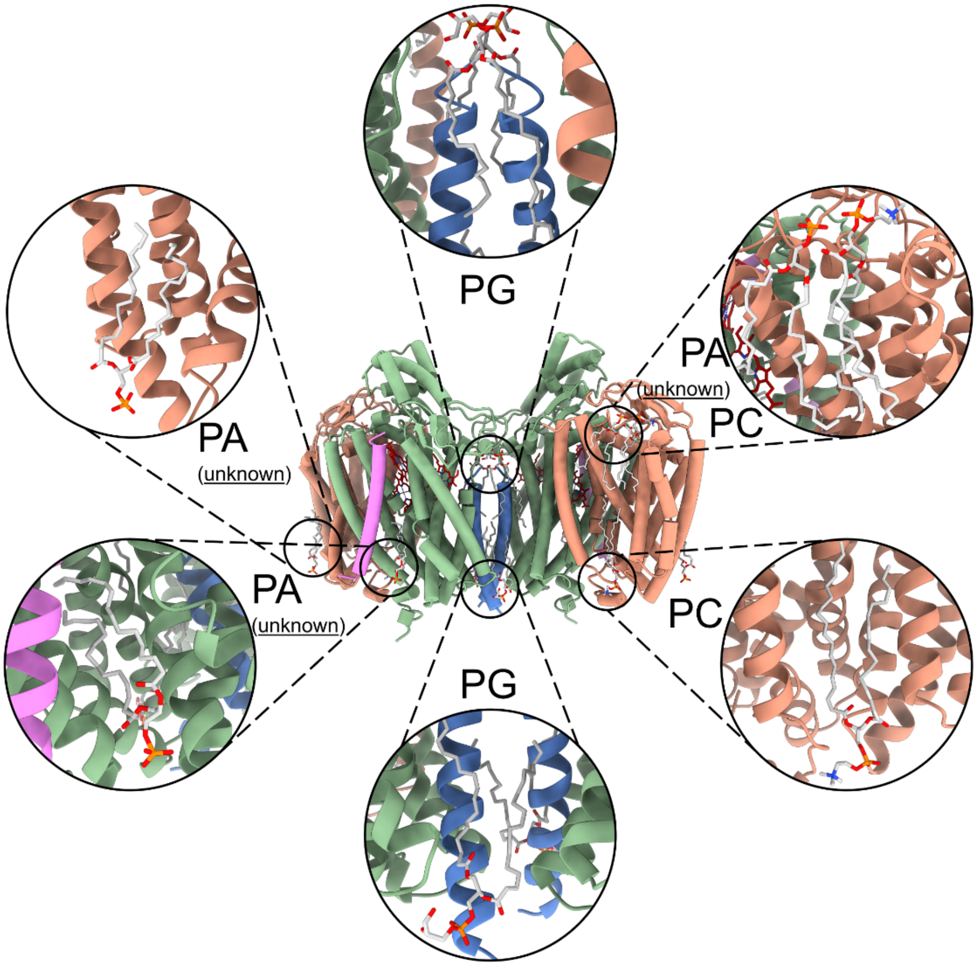

Fig. S8. Lipids bound peripherally and to the interface of the *Ecbd* dimer. Lipids with unclear headgroup densities are modelled as phosphatidic acid (PA).

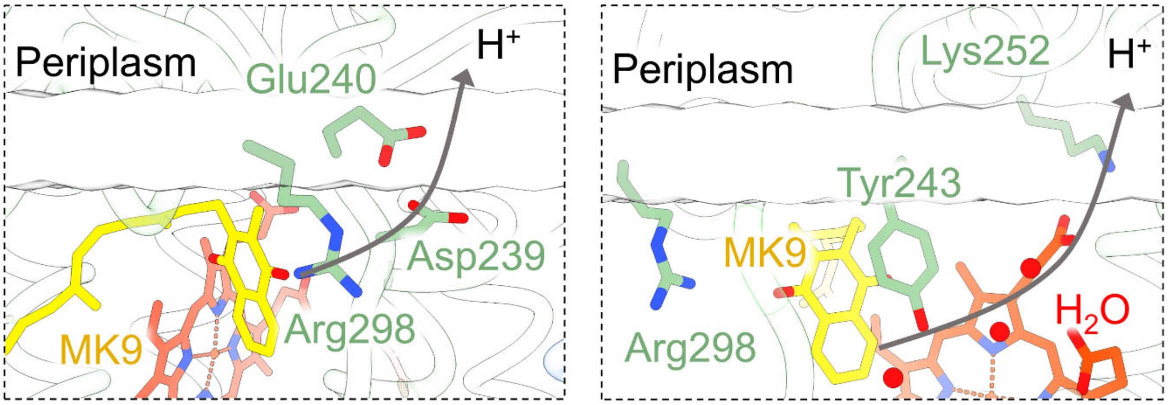

Fig. S9. Putative proton transfer routes from the quinol oxidation site towards the periplasm. One route transfers past the Asp239^{CydA} Glu240^{CydA} pair near the quinol oxidation site. The other route transfers via structures water molecules towards Lys252^{CydA} at the membrane interface.

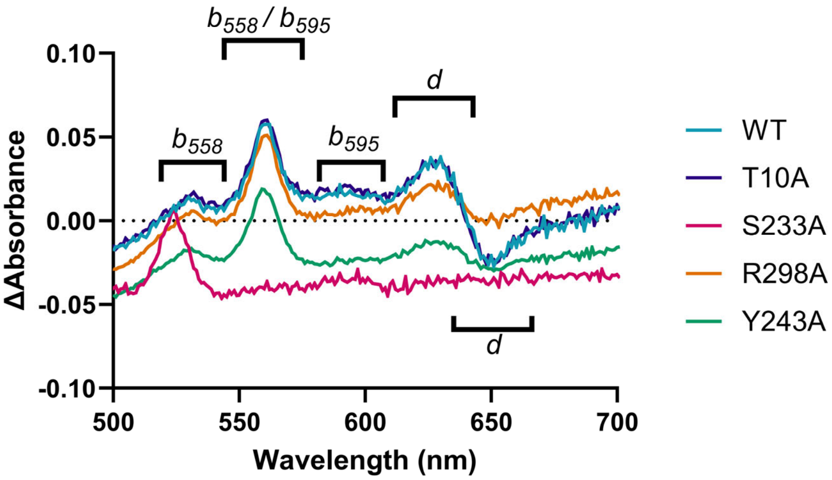

Fig. S10. Reduced minus oxidized spectra of the *Ecbd* WT and the Y234A, R298A, T10A, S233A mutants. The data was normalized to the Soret band (415 nm) of the oxidized spectrum.

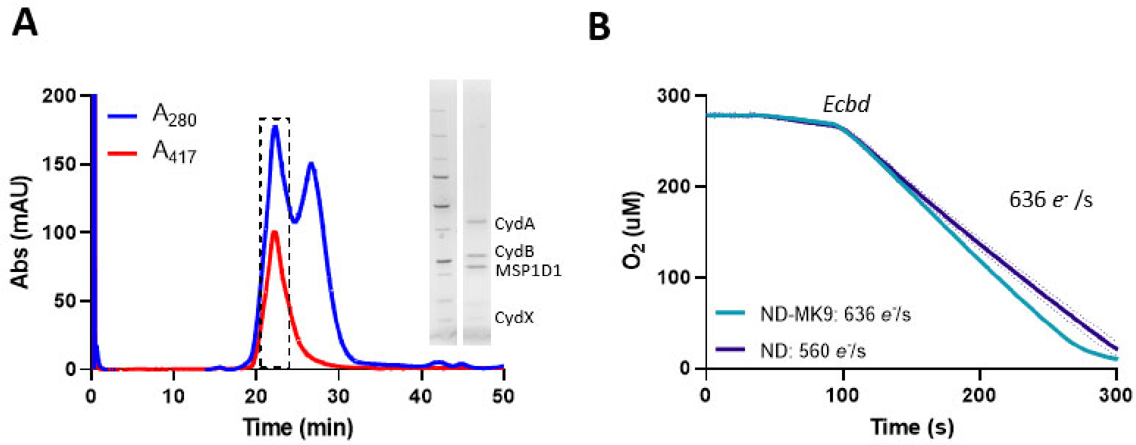

Fig. S11. *Ecbd* reconstitution in nanodiscs. (**A**) Size exclusion of *Ecbd* nanodiscs. (**B**) Activity of *Ecbd* nanodiscs with either MK or UQ.

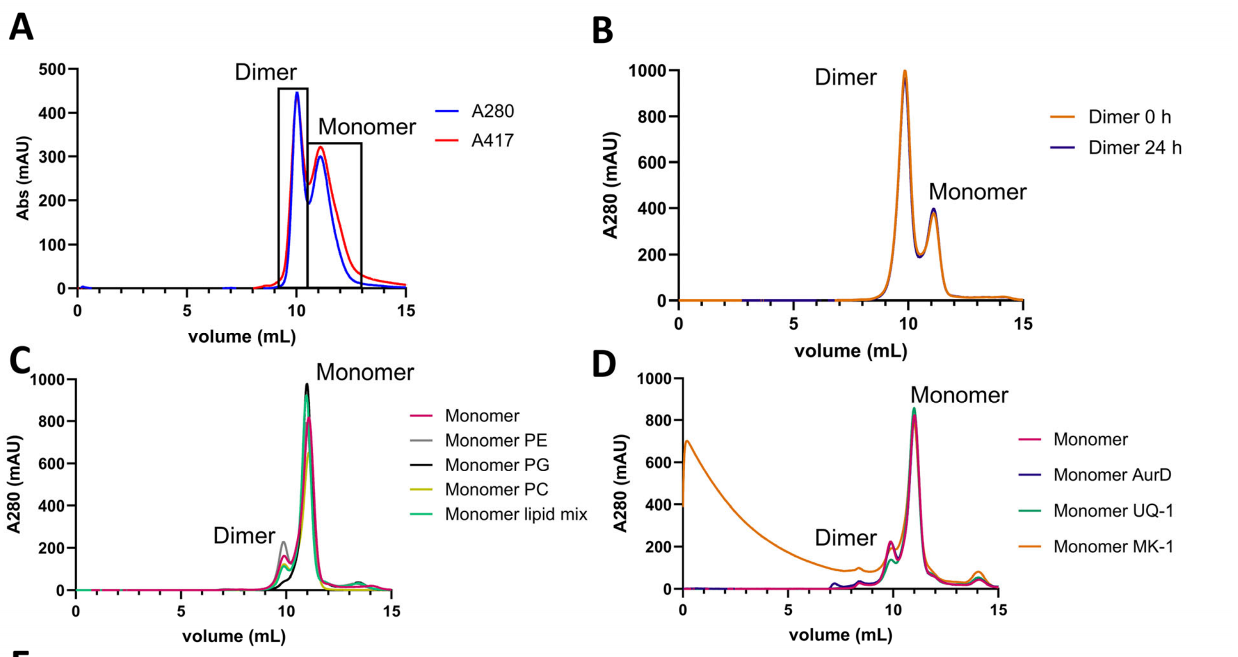

E

	Dimer peak Mw (kDa)	Monomer peak Mw (kDa)
Dimer 0 h	379.5	222.2
Dimer 24 h	380.4	216.1
Monomer	346.4	215.9
Monomer_Lipid Mix	407.9	220.8
Monomer_PE	669.9	269.4
Monomer_PG	-	215.5
Monomer AurD	865.6	296.3
Monomer UQ-1	368.8	227.5
Monomer MK-1	467.7	240.6

Fig. S12. SEC-(MALS) analysis of the *Ecbd* monomer and dimer. (**A**) SEC of the Strep purified *Ecbd* in LMNG on a superdex 200 column, enriching monomer and dimer *Ecbd* samples. (**B**) Stability of the SEC-purified *Ecbd* dimer (boxed in panel (A)) in detergent on SEC MALS. (**C**) SEC-MALS traces of SEC-purified *Ecbd* monomers (boxed in panel (A)) showing stable oligomeric state upon incubates with lipids or (**D**) incubation with quinones and Aurachin D (AurD). The addition of MK results in a decreasing baseline due to its absorption at 280 nm. (**E**) Mass determination by SEC MALS of the monomeric and dimeric *Ecbd* peaks.

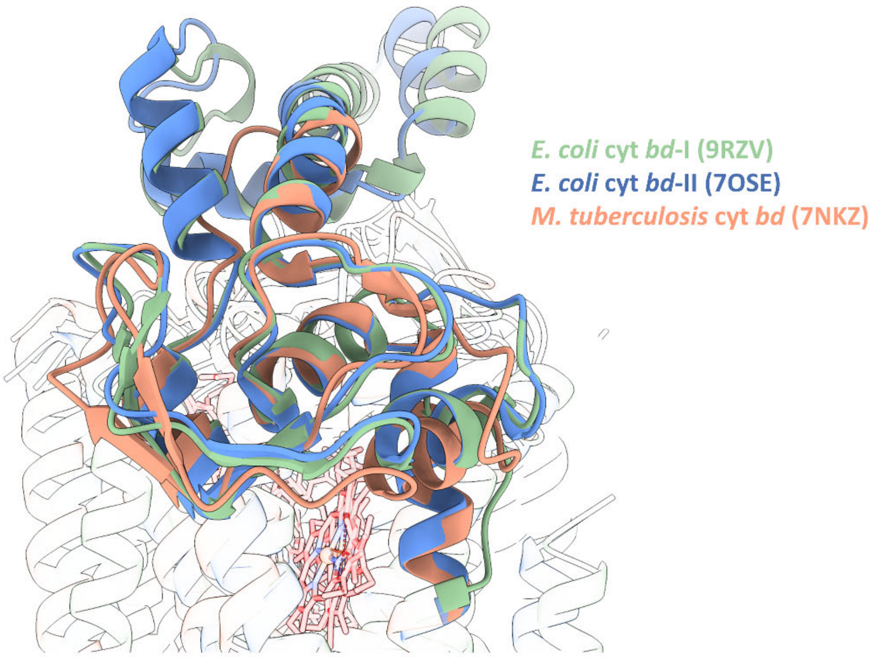

Fig. S13. Overlay of the Q-loop fold found in the *E. coli* cyt *bd*-I MK bound state (9RZV), *E. coli* cyt *bd*-I AurD bound state (7OSE), and *M. tuberculosis* cyt *bd*.

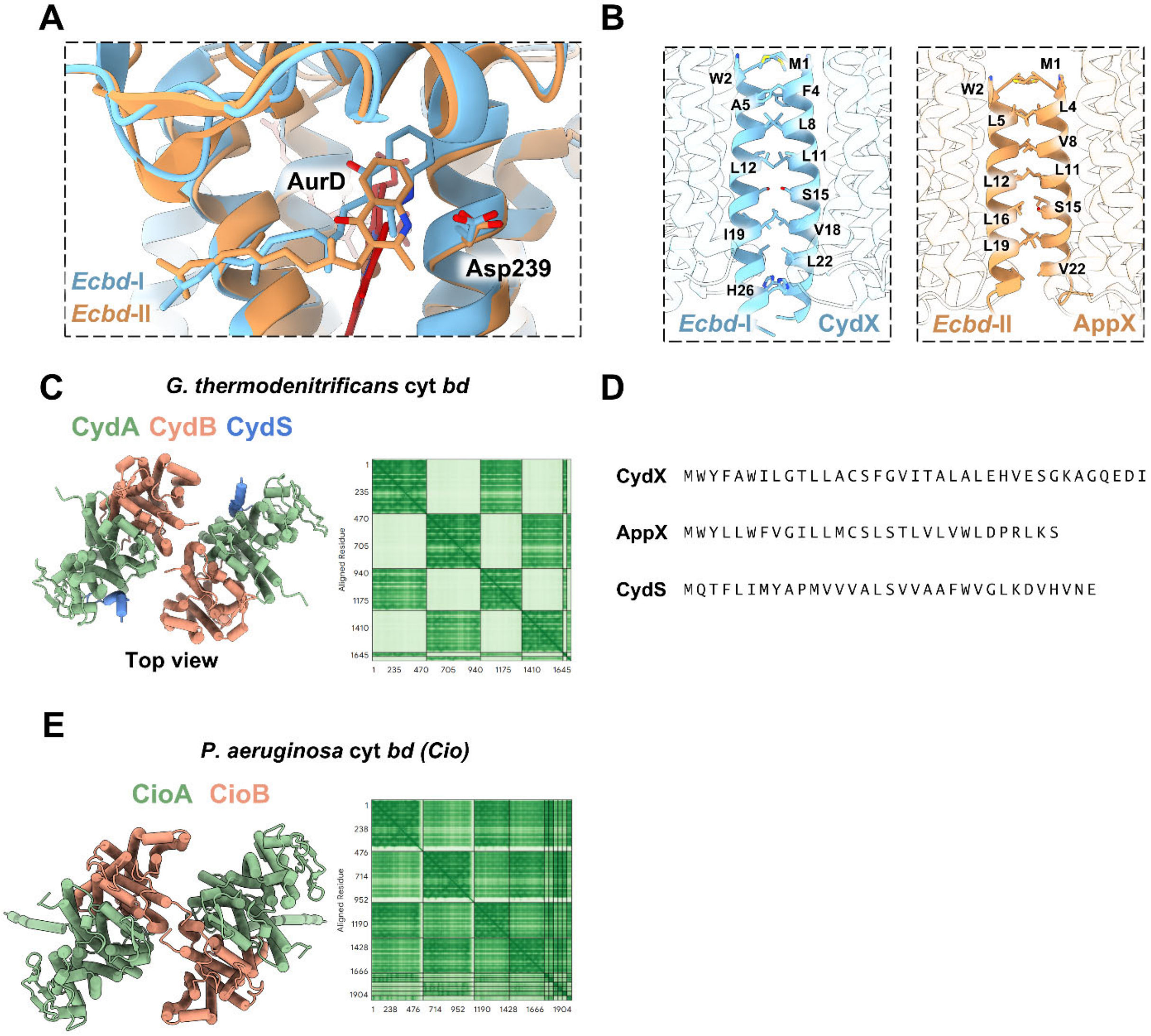

Fig. S14. Comparison of AurD binding and the dimer interface (**A**) binding of AurD to *Ecbd*-I and *Ecbd*-II (7OSE) (**B**) Dimer interface of *Ecbd*-I CydX and *Ecbd*-II AppX (**C**) Predicted *G. thermodenitrificans* cyt *bd* dimer using alphafold 3, indicating Cyd S does not stabilize dimer formation. (**D**) sequence comparison of *E. coli* CydX, AppX and *G. thermodenitrificans* CydS. (**E**) Predicted *P. aeruginosa* cyt *bd* dimer using alphafold 3, indicating dimer formation formation via interactions of CydB.

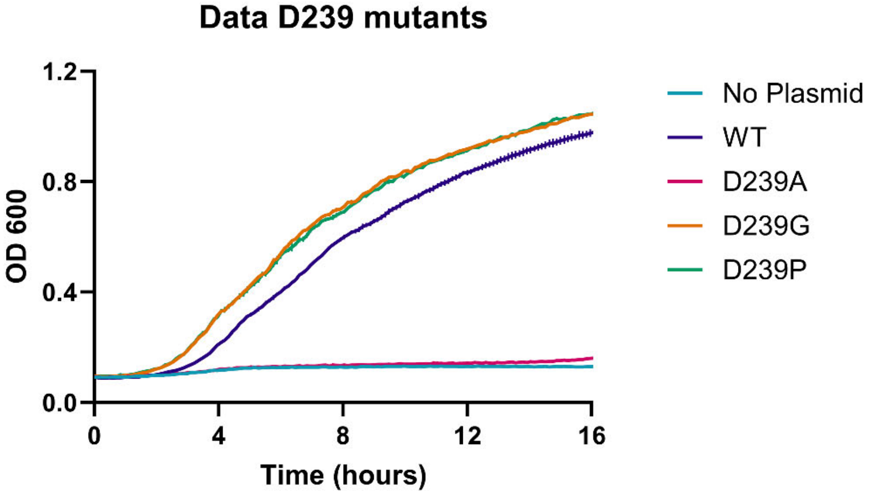

Fig. S15. Growth curves of the MB43 ΔcydA knockout strain supplemented with *Ecbd* WT or its D239^{CydA} mutants.

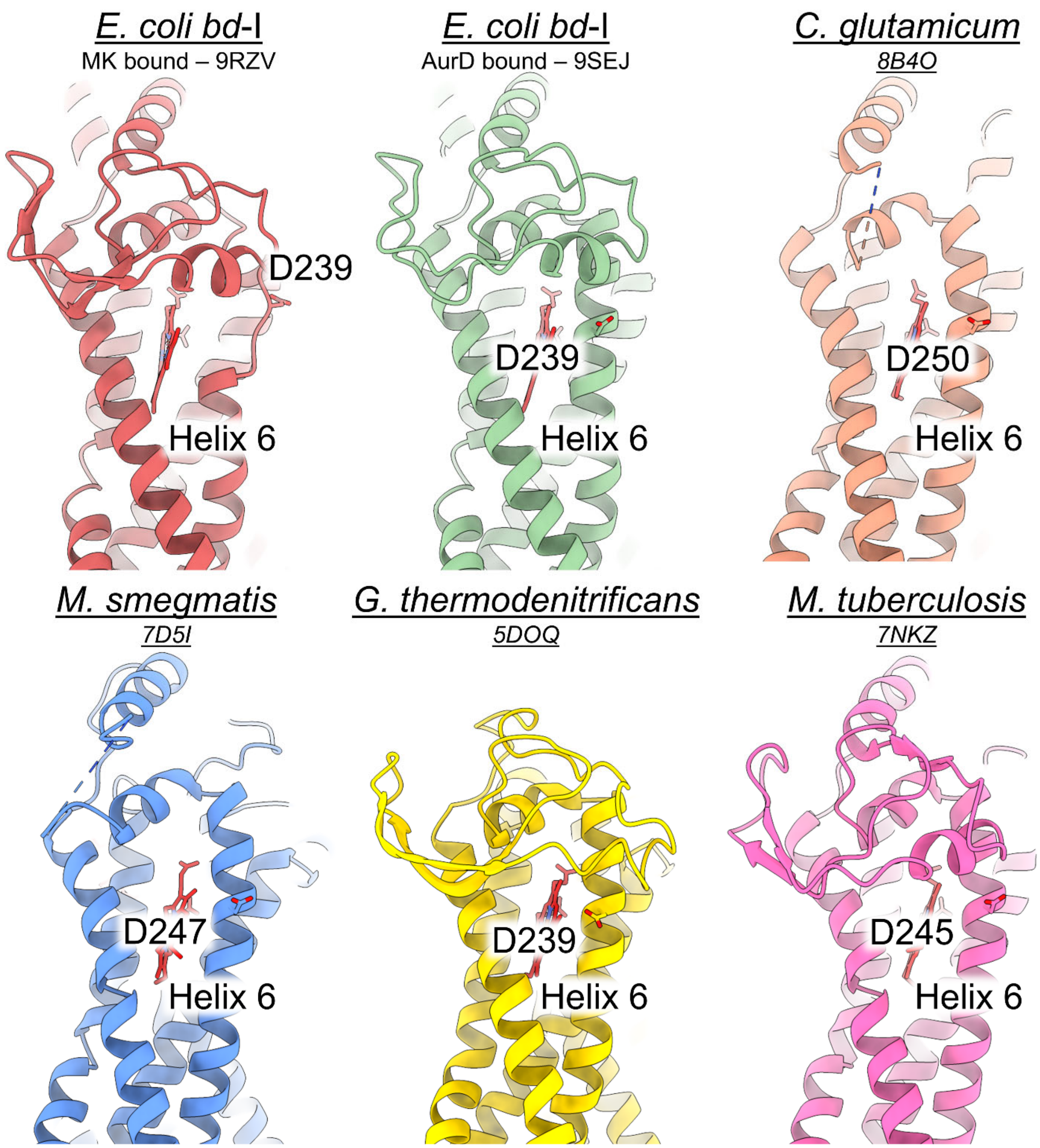

Fig. S16. Overview of the helical states of cyt *bd* oxidases. The short Q-loop *bd* oxidases represent the helical fold of helix 6, as in the AurD inhibited state of *E. coli* cytochrome *bd*-I

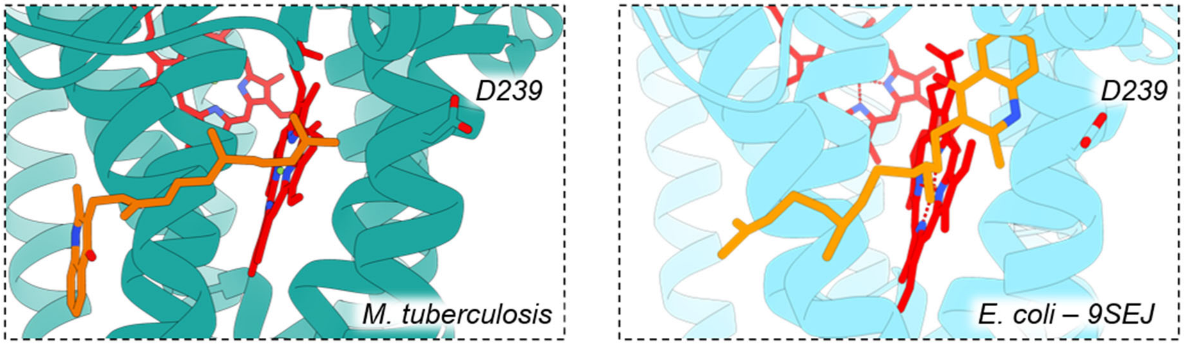

Fig. S17. Comparison of the predicted binding pose of Aurachin D in *M. tuberculosis* cytochrome *bd* using AutoDock Vina *(54, 55)* via Swissdock *(56, 57)*, with the cryo-EM structure of *Ecbd* bound to Aurachin D (9SEJ).

Table S1. Cryo-EM data collection, refinement and validation statistics

	***Ecbd* monomer apo (EMDB-54823) (PDB 9SFH)**	***Ecbd* monomer MK incubated (EMDB-54822) (PDB 9SFF)**	***Ecbd* monomer AurD incubated (EMDB-54801) (PDB 9SE4)**	***Ecbd* dimer MK bound Closed (EMDB-54414) (PDB 9RZV)**	***Ecbd* dimer MK bound-open/closed (EMDB-54826) (PDB 9SFJ)**	***Ecbd* dimer unbound/MK closed (EMDB-54866) (PDB 9SFV)**	***Ecbd* dimer AurD (EMDB-54812) (PDB 9SEJ)**
**Data collection and processing**							
Magnification	130,000	105,000	130,000	105,000	130,000	130,000	130,000
Voltage (kV)	200	300	200	300	200	200	200
Electron exposure (e–/Å^2)	100	100	100	100	100	100	100
Defocus range (μm)	-0.8 - -2.0	-1.0 - -2.2	-0.8 - -2.0	-1.0 - -2.2	-0.8 - -2.0	-0.8 - -2.0	-0.8 - -2.0
Pixel size (Å)	0.880	0.836	0.880	0.836	0.880	0.880	0.880
Symmetry imposed	C1	C1	C1	C2	C1	C1	C2
Initial particle images (no.)	6,873,607	2,979,789	3,721,318	3,289,126	6,873,607	6,873,607	2,009,440
Final particle images (no.)	764,760	380,310	399,575	530,094	224,083	152,474	255,127
Map resolution (Å)	2.49	2.39	2.47	2.23	2.52	2.61	2.47
FSC threshold	0.143	0.143	0.143	0.143	0.143	0.143	0.143
Map resolution range (Å)	2.46-2.6	2.39-2.51	2.47-2.56	2.21-2.28	2.52-2.58	2.5-2.56	2.45-2.51
**Refinement**							
Initial model used (PDB code)		6RKO		Alphafold			
Model resolution (Å)	2.6	2.4	2.5	2.2	2.4	2.5	2.3
FSC threshold	0.5	0.5	0.5	0.5	0.5	0.5	0.5
Map sharpening *B* factor (Å^2)	-60	-60	-60	-40	-60	-60	-60
**Model composition**							
Non-hydrogen atoms	7492	7492	7492	16307	15464	15412	15975
Protein residues	892	892	892	1902	1902	1858	1902
Ligands	2 HEM, 1 A1JN4, 3 LPP, 1 UQ8, 1 OXY	2 HEM, 1 A1JN4, 3 LPP, 1 MQ9, 2 POV, 1 OXY	2 HEM, 1 A1JN4, 3 LPP, 1 UQ8, 1 OXY	4 HEM, 2 A1JN4, 6 LPP, 4 MQ9, 4 PGT, 4 POV, 2 UQ8, 2 OXY	4 HEM, 2 A1JN4, 6 LPP, 2 MQ8, 4 UQ8, 2 OXY	4 HEM, 2 A1JN4, 6 LPP, 1 MQ8, 4 UQ8, 2 OXY	4 HEM, 2 A1JN4, 4 LPP, 2 0NI, 2 PGT, 2 POV, 4 UQ8, 2 OXY
Water	32	32	32	77	10	10	98
B factors (Å^2)							
Protein	68.40	79.60	63.40	53.92	97.41	124.62	78.28

Ligand	52.36	87.37	50.36	62.80	100.32	122.41	77.50
**R.m.s. deviations**							
Bond lengths (Å)	0.003	0.003	0.003	0.004	0.004	0.004	0.004
Bond angles (°)	0.805	0.703	0.806	0.749	1.460	1.140	1.47
**Validation**							
MolProbity score	1.60	1.39	1.58	1.31	1.40	1.32	1.37
Clashscore	7.79	5.68	7.51	2.01	5.05	2.29	4.28
Poor rotamers (%)	1.5	0.55	1.64	0.26	1.32	1.12	0.58
**Ramachandran plot**							
Favored (%)	97.85	97.84	97.96	99.26	97.83	97.61	97.93
Allowed (%)	2.04	2.16	2.04	0.96	2.17	2.39	1.96
Disallowed (%)	0.11	0	0	0.05	0	0	0.11

Table S2. Conservation of quinol binding interactions in CydA across the *bd* oxidase family

Ecbd	**S233**	**Y243**	**R298**
qOR1	L 42%<br>S 23%<br>A 16%<br>G 10%<br>F 4%	Y 31%<br>R 20%<br>K 19%<br>Q 13 %<br>E 4%	R 40%<br>G 26%<br>N 13%<br>H 10%<br>D 2%
qOR1-Long	S 64%<br>L 12%<br>G 9%<br>A 6%<br>F 6%	Y 91%<br>Q 3%<br>R 3%<br>V 3%	R 82%<br>H 15%<br>G 3%
qOR1-Short	L 57%<br>A 21%<br>G 11%<br>F 3%<br>V 3%	K 29%<br>R 29%<br>Q 17%<br>E 6%<br>V 5%	G 38%<br>N 19%<br>R 17%<br>H 8%<br>D 3%
qOR2	L 61%<br>F 14%<br>I 12%<br>A 4%<br>P 4%	R 39%<br>K 33%<br>Q 16%<br>E 4%<br>H 4%	G 29%<br>H 16%<br>D 14%<br>N 14%<br>F 10%
qOR3	A 34%<br>L 22%<br>T 17%<br>G 15%<br>I 5%	K 49%<br>Q 29%<br>I 17%<br>E 2%<br>R 2%	N 44%<br>G 27%<br>L 15%<br>D 5%<br>H 2%
qOR4a	I 58%<br>A 17%<br>Q 13%<br>V 13%	E 79%<br>D 21%	G 88%<br>L 12%
qOR4b	L 34%<br>I 23%<br>A 19%<br>P 17%<br>V 6%	Y 36%<br>V 15%<br>A 11%<br>M 11%<br>L 9%	D 6%<br>G 6%<br>Q 4%<br>F 2%<br>P 2%

Table S3. Conservation of AurD interactions in CydA across the *bd* oxidase family.

Ecbd	**D239**	**G242**	**Y243**	**E257**	**I295**	**A296**	**F390**	**R391**	**V394**
**qOR1**	D 66%<br>H 30%<br>S 2%	S 54%<br>Q 23%<br>H 8%<br>T 6%<br>L 3%	Y 31%<br>R 20%<br>K 19%<br>Q 13%<br>E 4%	E 99%<br>Q 1%	L 64%<br>I 26%<br>M 6%<br>F 2%<br>V 2%	A 46%<br>T 45%<br>V 14%<br>G 10%<br>L 5%	F 69%<br>Y 30%<br>M 1%	R 56%<br>H 44%	V 67%<br>I 26%<br>A 6%<br>F 1%
**qOR1-Long**	D 97%<br>H 3%	G 82%<br>A 18%	Y 91%<br>Q 3%<br>R 3%<br>V 3%	E 100%	I 70%<br>L 24%<br>M 3%<br>V 3%	A 61%<br>G 24%<br>L 6%<br>T 6%<br>S 3%	F 100%	R 88%<br>H 12%	V 100%
**qOR1-Short**	D 52%<br>H 44%<br>S 3%	A 67%<br>G 24%<br>S 3%<br>I 2%<br>M 2%	K 29%<br>R 29%<br>Q 17%<br>E 6%<br>V 5%	E 98%<br>Q 2%	L 84%<br>M 8%<br>F 3%<br>I 3%<br>V 2%	A 38%<br>V 21%<br>T 19%<br>S 6%<br>I 5%	F 52%<br>Y 46%<br>M 2%	H 60%<br>R 40%	V 49%<br>I 40%<br>A 10%<br>F 2%
**qOR2**	D 73%<br>H 27%	A 84%<br>G 12%<br>S 4%	R 39%<br>K 33%<br>Q 16%<br>E 4%<br>H 4%	E 100%	L 75%<br>M 16%<br>I 6%<br>V 4%	A 69%<br>L 18%<br>V 10%<br>S 2%<br>T 2%	F 100%	Q 75%<br>D 24%<br>E 2%	V 88%<br>I 6%<br>L6%
**qOR3**	D 54%<br>H 46%	A 56%<br>G 44%	K 49%<br>Q 29%<br>I 17%<br>E 2%<br>R 2%	E 100%	L 100%	A 85%<br>V 15%<br>G 2%	F 100%	D 51%<br>N 49%	V 93%<br>A 5%<br>I 2%
**qOR4a**	H 100%	G 100%	E 79%<br>D 21%	E 75%<br>T 25%	V 33%<br>F 29%<br>L 17%<br>P 13%<br>M 4%	A 71%<br>S 8%<br>T 8%<br>V 8%<br>Q 4%	Y 79%<br>F 13%<br>M 8%	Y 54%<br>L 21%<br>W 13%	V 75%<br>I 25%
**qOR4b**	A 23%<br>P 23%<br>G 21%<br>V 17%<br>F 3%	L 32%<br>A 17%<br>W 15%<br>F 11%<br>Y 8%	Y 36%<br>V15%<br>A 11%<br>M 11%<br>L 9%	T 8%<br>N 4%	L 8%<br>A 4%<br>S 4%	L 9%<br>I 4%<br>V 4%<br>F 2%	F 58%<br>L 17%<br>A 11%<br>I 8%<br>M 2%	Y 40%<br>V 19%<br>A11%<br>I 11%<br>L 9%	I 40%<br>V 38%<br>L 13%<br>M 4%<br>T 4%

Table S4. Mutagenesis primers

**Primer**	**Sequence**
Ecbd.A S233A Fw	atggctgctgttctggctgttattgttctgggt
Ecbd.A S233A Rv	acccagaacaataacagccagaacagcagccat
Ecbd.A D239A Fw	tctgttattgttctgggtgccgaatccggctacgaaatgggc
Ecbd.A D239A Rv	gcccatttcgtagccggattcggcacccagaacaataacaga
Ecbd.A D239G Fw	tctgttattgttctgggtggcgaatccggctacgaaatgggc
Ecbd.A D239G Rv	gcccatttcgtagccggattcgccacccagaacaataacaga
Ecbd.A D239P Fw	attgttctgggtccagaatccggctac
Ecbd.A D239P Rv	gtagccggattctggacccagaacaat
Ecbd.A Y243A Fw	ctgggtgacgaatccggcgccgaaatgggcgacgtgcag
Ecbd.A Y243A Rv	ctgcacgtcgcccatttcggcgccggattcgtcacccag
Ecbd.A R298A Fw	ctgggcatcattgcaacggcatccgtggataccccggtt
Ecbd.A R298A Rv	aaccggggtatccacggatgccgttgcaatgatgcccag
Ecbd.X T10A Fw	gcatggattctgggagctcttcttgcctgttcg
Ecbd.X T10A Rv	cgaacaggcaagaagagctcccagaatccatgc
Ecbd Gibson Fwd	gcccgaaaggaagctgagtt
Ecbd Gibson Rev	aactcagcttcctttcgggc

REFERENCES

1. T. Friedrich, D. Wohlwend, V. B. Borisov, Recent advances in structural studies of cytochrome *bd* and its potential application as a drug target. *Int. J. Mol. Sci.* **23**, 3166 (2022).

2. E. Cox, K. Laessig, FDA approval of bedaquiline—The benefit–risk balance for drug-resistant tuberculosis. *N. Engl. J. Med.* **371**, 689–691 (2014).

3. R. D'Mello, S. Hill, R. K. Poole, The cytochrome bd quinol oxidase in Escherichia coli has an extremely high oxygen affinity and two oxygen-binding haems: Implications for regulation of activity in vivo by oxygen inhibition. *Microbiology* **142**, 755–763 (1996).

4. I. Belevich, V. B. Borisov, D. A. Bloch, A. A. Konstantinov, M. I. Verkhovsky, Cytochrome *bd* from *Azotobacter vinelandii*: Evidence for high-affinity oxygen binding. *Biochemistry* **46**, 11177–11184 (2007).

5. M. G. Mason, M. Shepherd, P. Nicholls, P. S. Dobbin, K. S. Dodsworth, R. K. Poole, C. E. Cooper, Cytochrome *bd* confers nitric oxide resistance to *Escherichia coli*. *Nat. Chem. Biol.* **5**, 94–96 (2009).

6. X. Xia, S. Wu, L. Li, B. Xu, G. Wang, The cytochrome *bd* complex is essential for chromate and sulfide resistance and is regulated by a GbsR-type regulator, CydE, in *Alishewanella* sp. WH16–1. *Front. Microbiol.* **9**, 1849 (2018).

7. C. J. Beebout, L. A. Sominsky, A. R. Eberly, G. T. Van Horn, M. Hadjifrangiskou, Cytochrome *bd* promotes *Escherichia coli* biofilm antibiotic tolerance by regulating accumulation of noxious chemicals. *NPJ Biofilms Microbiomes* **7**, 35 (2021).

8. L. Mascolo, D. Bald, Cytochrome *bd* in *Mycobacterium tuberculosis*: A respiratory chain protein involved in the defense against antibacterials. *Prog. Biophys. Mol. Biol.* **152**, 55–63 (2020).

9. B. S. Lee, K. Hards, C. A. Engelhart, E. J. Hasenoehrl, N. P. Kalia, J. S. Mackenzie, E. Sviriaeva, S. M. S. Chong, M. S. S. Manimekalai, V. H. Koh, J. Chan, J. Xu, S. Alonso, M. J. Miller, A. J. C. Steyn, G. Grüber, D. Schnappinger, M. Berney, G. M. Cook, G. C. Moraski, K. Pethe, Dual

inhibition of the terminal oxidases eradicates antibiotic-tolerant *Mycobacterium tuberculosis* . *EMBO Mol. Med.* **13**, e13207 (2021).

10. J. Jones-Carson, M. Husain, L. Liu, D. J. Orlicky, A. Vázquez-Torres, Cytochrome bd-dependent bioenergetics and antinitrosative defenses in Salmonella pathogenesis. *MBio* **7**, e02052-16 (2016).

11. M. Shepherd, M. E. S. Achard, A. Idris, M. Totsika, M. D. Phan, K. M. Peters, S. Sarkar, C. A. Ribeiro, L. V. Holyoake, D. Ladakis, G. C. Ulett, M. J. Sweet, R. K. Poole, A. G. McEwan, M. A. Schembri, The cytochrome *bd*-I respiratory oxidase augments survival of multidrug-resistant *Escherichia coli* during infection. *Sci. Rep.* **6**, 35285 (2016).

12. S. A. Henry, C. M. Webster, L. N. Shaw, N. J. Torres, M. E. Jobson, B. C. Totzke, J. K. Jackson, J. E. McGreig, M. N. Wass, G. K. Robinson, M. Shepherd, Steroid drugs inhibit bacterial respiratory oxidases and are lethal toward methicillin-resistant *Staphylococcus aureus*. *J. Infect. Dis.* **230**, e149–e158 (2024).

13. S. Safarian, A. Hahn, D. J. Mills, M. Radloff, M. L. Eisinger, A. Nikolaev, J. Meier-Credo, F. Melin, H. Miyoshi, R. B. Gennis, J. Sakamoto, J. D. Langer, P. Hellwig, W. Kühlbrandt, H. Michel, Active site rearrangement and structural divergence in prokaryotic respiratory oxidases. *Science* **366**, 100–104 (2019).

14. S. Safarian, C. Rajendran, H. Müller, J. Preu, J. D. Langer, S. Ovchinnikov, T. Hirose, T. Kusumoto, J. Sakamoto, H. Michel, Structure of a bd oxidase indicates similar mechanisms for membrane integrated oxygen reductases. *Science* **352**, 583–586 (2016).

15. R. Murali, R. B. Gennis, J. Hemp, Evolution of the cytochrome bd oxygen reductase superfamily and the function of CydAA’ in Archaea. *ISME J.* **15**, 3534–3548 (2021).

16. H. G. Goojani, J. Konings, H. Hakvoort, S. Hong, R. B. Gennis, J. Sakamoto, H. Lill, D. Bald, The carboxy-terminal insert in the Q-loop is needed for functionality of *Escherichia coli* cytochrome bd-I. *Biochim. Biophys. Acta Bioenerg.* **1861**, 148175 (2020).

17. A. Theßeling, S. Burschel, D. Wohlwend, T. Friedrich, The long Q-loop of *Escherichia coli* cytochrome *bd* oxidase is required for assembly and structural integrity. *FEBS Lett.* **594**, 1577–1585 (2020).

18. G. Unden, J. Bongaerts, Alternative respiratory pathways of *Escherichia coli*: Energetics and transcriptional regulation in response to electron acceptors. *Biochim. Biophys. Acta* **1320**, 217–234 (1997).

19. A. Theßeling, T. Rasmussen, S. Burschel, D. Wohlwend, J. Kägi, R. Müller, B. Böttcher, T. Friedrich, Homologous *bd* oxidases share the same architecture but differ in mechanism. *Nat. Commun.* **10**, 5138 (2019).

20. T. N. Grund, M. Radloff, D. Wu, H. G. Goojani, L. F. Witte, W. Jösting, S. Buschmann, H. Müller, I. Elamri, S. Welsch, H. Schwalbe, H. Michel, D. Bald, S. Safarian, Mechanistic and structural diversity between cytochrome bd isoforms of *Escherichia coli*. *Proc. Natl. Acad. Sci. U.S.A.* **118**, e2114013118 (2021).

21. A. Grauel, J. Kägi, T. Rasmussen, I. Makarchuk, S. Oppermann, A. F. A. Moumbock, D. Wohlwend, R. Müller, F. Melin, S. Günther, P. Hellwig, B. Böttcher, T. Friedrich, Structure of *Escherichia coli* cytochrome *bd*-II type oxidase with bound aurachin D. *Nat. Commun.* **12**, 6498 (2021).

22. R. Timkovich, M. S. Cork, R. B. Gennis, P. Y. Johnson, Proposed structure of heme d, a prosthetic group of bacterial terminal oxidases. *J. Am. Chem. Soc.* **107**, 6069–6075 (1985).

23. T. T. van der Velden, K. Kayastha, C. Y. J. Waterham, S. Brünle, L. J. C. Jeuken, Menaquinone-specific turnover by *Mycobacterium tuberculosis* cytochrome *bd* is redox regulated by the Q-loop disulfide bond. *J. Biol. Chem.* **301**, 108094 (2025).

24. L. J. C. Jeuken, S. D. Connell, M. Nurnabi, J. O'Reilly, P. J. F. Henderson, S. D. Evans, R. J. Bushby, Direct electrochemical interaction between a modified gold electrode and a bacterial membrane extract. *Langmuir* **21**, 1481–1488 (2005).

25. Y. Nakatani, Y. Shimaki, D. Dutta, S. P. Muench, K. Ireton, G. M. Cook, L. J. C. Jeuken, Unprecedented properties of phenothiazines unraveled by a NDH-2 bioelectrochemical assay platform. *J. Am. Chem. Soc.* **142**, 1311–1320 (2020).

26. S. A. Weiss, R. J. Bushby, S. D. Evans, L. J. C. Jeuken, A study of cytochrome bo3 in a tethered bilayer lipid membrane. *Biochim. Biophys. Acta Bioenerg.* **1797**, 1917–1923 (2010).

27. A. Godoy-Hernandez, A. H. Asseri, A. J. Purugganan, C. Jiko, C. de Ram, H. Lill, M. Pabst, K. Mitsuoka, C. Gerle, D. Bald, D. G. G. McMillan, Rapid and highly stable membrane reconstitution by LAiR enables the study of physiological integral membrane protein functions. *ACS Cent. Sci.* **9**, 494–507 (2023).

28. L. J. C. Jeuken, S. D. Connell, P. J. F. Henderson, R. B. Gennis, S. D. Evans, R. J. Bushby, Redox enzymes in tethered membranes. *J. Am. Chem. Soc.* **128**, 1711–1716 (2006).

29. L. Pravda, D. Sehnal, D. Toušek, V. Navrátilová, V. Bazgier, K. Berka, R. S. Vařeková, J. Koča, M. Otyepka, MOLEonline: A web-based tool for analyzing channels, tunnels and pores (2018 update). *Nucleic Acids Res.* **46**, W368–W373 (2018).

30. M. Janczak, J. Vilhjálmsdóttir, P. Ädelroth, Proton transfer in cytochrome bd-I from *E. coli* involves Asp-105 in CydB. *Biochim. Biophys. Acta Bioenerg.* **1865**, 149046 (2024).

31. S. Safarian, H. K. Opel-Reading, D. Wu, A. R. Mehdipour, K. Hards, L. K. Harold, M. Radloff, I. Stewart, S. Welsch, G. Hummer, G. M. Cook, K. L. Krause, H. Michel, The cryo-EM structure of the *bd* oxidase from *M. tuberculosis* reveals a unique structural framework and enables rational drug design to combat TB. *Nat. Commun.* **12**, 5236 (2021).

32. M. Radloff, I. Elamri, T. N. Grund, L. F. Witte, K. F. Hohmann, S. Nakagaki, H. G. Goojani, H. Nasiri, H. Miyoshi, D. Bald, H. Xie, J. Sakamoto, H. Schwalbe, S. Safarian, Short-chain aurachin D derivatives are selective inhibitors of E. coli cytochrome bd-I and bd-II oxidases. *Sci. Rep.* **11**, 19906 (2021).

33. K. Yang, J. Zhang, A. S. Vakkasoglu, R. Hielscher, J. P. Osborne, J. Hemp, H. Miyoshi, P. Hellwig, R. B. Gennis, Glutamate 107 in subunit I of the cytochrome *bd* quinol oxidase from *Escherichia coli* is protonated and near the heme *d*/heme b_{595} binuclear center. *Biochemistry* **46**, 3270–3278 (2007).

34. R. J. Allen, E. P. Brenner, C. E. VanOrsdel, J. J. Hobson, D. J. Hearn, M. R. Hemm, Conservation analysis of the CydX protein yields insights into small protein identification and evolution. *BMC Genomics* **15**, 946 (2014).

35. J. Hoeser, S. Hong, G. Gehmann, R. B. Gennis, T. Friedrich, Subunit CydX of *Escherichia coli* cytochrome *bd* ubiquinol oxidase is essential for assembly and stability of the di-heme active site. *FEBS Lett.* **588**, 1537–1541 (2014).

36. C. E. VanOrsdel, S. Bhatt, R. J. Allen, E. P. Brenner, J. J. Hobson, A. Jamil, B. M. Haynes, A. M. Genson, M. R. Hemma, The *Escherichia coli* CydX protein is a member of the CydAB cytochrome *bd* oxidase complex and is required for cytochrome *bd* oxidase activity. *J. Bacteriol.* **195**, 3640–3650 (2013).

37. T. Mogi, S. Akimoto, S. Endou, T. Watanabe-Nakayama, E. Mizuochi-Asai, H. Miyoshi, Probing the ubiquinol-binding site in cytochrome *bd* by site-directed mutagenesis. *Biochemistry* **45**, 7924–7930 (2006).

38. J. P. Richard, Protein flexibility and stiffness enable efficient enzymatic catalysis. *J. Am. Chem. Soc.* **141**, 3320–3331 (2019).

39. H. Arai, T. Kawakami, T. Osamura, T. Hirai, Y. Sakai, M. Ishii, Enzymatic characterization and in vivo function of five terminal oxidases in *Pseudomonas aeruginosa*. *J. Bacteriol.* **196**, 4206–4215 (2014).

40. W. Wang, Y. Gao, Y. Tang, X. Zhou, Y. Lai, S. Zhou, Y. Zhang, X. Yang, F. Liu, L. W. Guddat, Q. Wang, Z. Rao, H. Gong, Cryo-EM structure of mycobacterial cytochrome *bd* reveals two oxygen access channels. *Nat. Commun.* **12**, 4621 (2021).

41. T. N. Grund, Y. Kabashima, T. Kusumoto, D. Wu, S. Welsch, J. Sakamoto, H. Michel, S. Safarian, The cryoEM structure of cytochrome bd from *C. glutamicum* provides novel insights into structural properties of actinobacterial terminal oxidases. *Front. Chem.* **10**, 1085463 (2023).

42. M. Bekker, S. De Vries, A. Ter Beek, K. J. Hellingwerf, M. J. Teixeira De Mattos, Respiration of *Escherichia coli* can be fully uncoupled via the nonelectrogenic terminal cytochrome bd-II oxidase. *J. Bacteriol.* **191**, 5510–5517 (2009).

43. T. G. J. Knetsch, M. Ubbink, The effect of lipid composition on the thermal stability of nanodiscs. *Biochim. Biophys. Acta Biomembr.* **1866**, 184239 (2024).

44. N. Boden, R. J. Bushby, S. Clarkson, S. D. Evans, P. F. Knowles, A. Marsh, The design and synthesis of simple molecular tethers for binding biomembranes to a gold surface. *Tetrahedron* **53**, 10939–10952 (1997).

45. L. J. C. Jeuken, N. N. Daskalakis, X. Han, K. Sheikh, A. Erbe, R. J. Bushby, S. D. Evans, Phase separation in mixed self-assembled monolayers and its effect on biomimetic membranes. *Sens. Actuators B. Chem.* **124**, 501–509 (2007).

46. A. Punjani, J. L. Rubinstein, D. J. Fleet, M. A. Brubaker, cryoSPARC: Algorithms for rapid unsupervised cryo-EM structure determination. *Nat. Methods* **14**, 290–296 (2017).

47. A. Punjani, H. Zhang, D. J. Fleet, Non-uniform refinement: Adaptive regularization improves single-particle cryo-EM reconstruction. *Nat. Methods* **17**, 1214–1221 (2020).

48. J. L. Rubinstein, M. A. Brubaker, Alignment of cryo-EM movies of individual particles by optimization of image translations. *J. Struct. Biol.* **192**, 188–195 (2015).

49. J. Jumper, R. Evans, A. Pritzel, T. Green, M. Figurnov, O. Ronneberger, K. Tunyasuvunakool, R. Bates, A. Žídek, A. Potapenko, A. Bridgland, C. Meyer, S. A. A. Kohl, A. J. Ballard, A. Cowie, B. Romera-Paredes, S. Nikolov, R. Jain, J. Adler, T. Back, S. Petersen, D. Reiman, E. Clancy, M. Zielinski, M. Steinegger, M. Pacholska, T. Berghammer, S. Bodenstein, D. Silver, O. Vinyals, A. W. Senior, K. Kavukcuoglu, P. Kohli, D. Hassabis, Highly accurate protein structure prediction with AlphaFold. *Nature* **596**, 583–589 (2021).

50. P. Emsley, B. Lohkamp, W. G. Scott, K. Cowtan, Features and development of Coot. *Acta Crystallogr. D Biol. Crystallogr.* **66**, 486–501 (2010).

51. P. D. Adams, R. W. Grosse-Kunstleve, L.-W. Hung, T. R. Ioerger, A. J. McCoy, N. W. Moriarty, R. J. Read, J. C. Sacchettini, N. K. Sauter, T. C. Terwilliger, PHENIX: Building new software for automated crystallographic structure determination. *Acta Crystallogr. D Biol. Crystallogr.* **58**, 1948–1954 (2002).

52. E. F. Pettersen, T. D. Goddard, C. C. Huang, E. C. Meng, G. S. Couch, T. I. Croll, J. H. Morris, T. E. Ferrin, UCSF ChimeraX: Structure visualization for researchers, educators, and developers. *Protein Sci.* **30**, 70–82 (2021).

53. R. A. Laskowski, M. B. Swindells, LigPlot+: Multiple ligand–protein interaction diagrams for drug discovery. *J. Chem. Inf. Model.* **51**, 2778–2786 (2011).

54. J. Eberhardt, D. Santos-Martins, A. F. Tillack, S. Forli, AutoDock Vina 1.2.0: New docking methods, expanded force field, and python bindings. *J. Chem. Inf. Model.* **61**, 3891–3898 (2021).

55. O. Trott, A. J. Olson, AutoDock Vina: Improving the speed and accuracy of docking with a new scoring function, efficient optimization, and multithreading. *J. Comput. Chem.* **31**, 455–461 (2010).

56. M. Bugnon, U. F. Röhrig, M. Goullieux, M. A. S. Perez, A. Daina, O. Michielin, V. Zoete, SwissDock 2024: Major enhancements for small-molecule docking with attracting cavities and AutoDock Vina. *Nucleic Acids Res.* **52**, W324–W332 (2024).

57. A. Grosdidier, V. Zoete, O. Michielin, SwissDock, a protein-small molecule docking web service based on EADock DSS. *Nucleic Acids Res.* **39**, W270–W277 (2011).
